# A new acute scaphoid fracture assessment method: a reliability study of the ‘long axis’ measurement

**DOI:** 10.1186/s12891-018-2236-y

**Published:** 2018-08-29

**Authors:** Benjamin J. F. Dean, Nicholas D. Riley, Earl Robert McCulloch, Jennifer C. E. Lane, Amy Beth Touzell, Alastair J. Graham

**Affiliations:** 1 Nuffield Department of Orthopaedics, Rheumatology and Musculoskeletal Sciences (NDORMS), University of Oxford, Botnar Research Centre, Windmill road, Oxford, OX3 7LD UK; 2 Nuffield Orthopaedic Centre, Windmill road, Oxford, OX3 7LD UK; 3 Frankston Hospital, Frankston, VIC Australia; 4 Buckinghamshire Hospitals NHS Trust, High Wycombe Hospital, High Wycombe, Amersham, HP11 2TT UK

**Keywords:** Scaphoid, Fracture, Classification, Acute, Non-union

## Abstract

**Background:**

The aim of this study was to assess the inter observer and intra observer reliability of acute scaphoid fracture classification methods including a novel ‘long axis’ measurement, a simple method which we have developed with the aim of improving agreement when describing acute fractures.

**Methods:**

We identified sixty patients with acute scaphoid fractures at two centres who had been investigated with both plain radiographs and a CT (Computed Tomography) scan within 4 weeks of injury. The fractures were assessed by three observers at each centre using three commonly used classification systems and the ‘long axis’ method.

**Results:**

Inter observer reliability: based on X-rays the ‘long axis’ measurement demonstrated substantial agreement (Intraclass Correlation Coefficient (ICC) =0.76) and was significantly more reliable than the Mayo (*p* < 0.01), the most reliable of the established classification systems with moderate levels of agreement (kappa = 0.56). Intra observer reliability: the long axis measurement demonstrated almost perfect agreement whether based on X-ray (ICC = 0.905) or CT (ICC = 0.900).

**Conclusions:**

This study describes a novel pragmatic ‘long axis’ method for the assessment of acute scaphoid fractures which demonstrates substantial inter and intra observer reliability. The ‘long axis’ measurement has clear potential benefits over traditional classification systems which should be explored in future clinical research.

## Background

Scaphoid fractures represent around 2–3% of all fractures and around 10% of all fractures in the hand, while the younger population is more typically affected although fractures do occur in the elderly [1]. Fractures of the mid-portion of the scaphoid, the so-called ‘waist’, are the most common [1]. The existing evidence suggests that the risk non-union is considerably higher for more proximal fractures [2]. Despite large numbers of publications on outcomes of scaphoid fracture management there are large inconsistencies in the published data. Combining these data groups is notoriously difficult for a number of reasons including variable demographics, inconsistent definitions of fracture type, and inconsistent methodology for defining outcome. There is particular interest in the behaviour of proximal pole fractures but no consensus on how this subgroup should be defined. This method could be used to give more reliable and reproducible descriptions of fracture type.

A number of classification systems have been described, with the most widely used being the Herbert, Russe and Mayo methods. However the reliability of these tools has been shown to be rather limited [3]. Despite the frequently-discussed distinction between proximal pole and waist fractures, there is no published reliable method of distinguishing between the two. The Mayo classification system divides the scaphoid into proximal, middle and distal third fractures, as well as distal tubercle and distal intra-articular fractures. Russe divided fractures into those with horizontal oblique, transverse or vertical oblique fracture lines [4]. The Herbert system divides acute fractures into either stable (Type A) and unstable (Type B), with various subdivisions with these types [5]. No study has described a method for determining precisely the location or the size of the proximal pole. For example the SWIFFT study protocol defines a proximal pole fracture as involving the ‘proximal fifth’ but does not describe how one can reliably determine when a fracture involves the proximal fifth [6]. This makes it difficult to compare studies and particularly difficult to perform meta-analysis on data from published cohorts. While the anatomical and radiological definition of proximal pole, waist and distal pole fractures is likely to remain contentious, looking at a more continuous measure of fracture site may give more clarity.

The primary aim of this study was to assess the reliability of acute scaphoid fracture assessment metrics including the new ‘long axis’ measurement. The secondary aims were to compare the reliability of this new method with three established classification systems, and to compare the reliability of each of these methods when using plain radiographs and CT. The null hypothesis was that there would be no difference in reliability between the older methods and the new ‘long axis’ measurement.

## Methods

Using local surgical databases we retrospectively identified sixty patients with acute scaphoid fractures across two centres that had been investigated with both plain radiographs and a CT scan within 4 weeks of injury. We excluded non acute scaphoid fractures as well as those associated with acute carpal dislocation. All injuries were sustained from the beginning of 2013 to the end of 2016.

The patient demographics were recorded. Two senior surgical trainees and an experienced hand surgeon analysed the plain radiographs and CT scans in each centre. The observers were briefed on recent literature which describes the long axis passing from the proximal point through the centre of the waist to the most distal point, with the most distal point being very close to the centre of the tubercle just radial to its apex [7, 8].

The Classification according to Russe, Herbert and Mayo systems were recorded. In addition the observers recorded the long axis length of the scaphoid, the distance at which the fracture line crossed the long axis, distance at which the proximal fracture line crossed a line perpendicular to long axis on ulnar border, distance at which fracture line crossed a line perpendicular to long axis on radial border, presence of a sagittal plane deformity, presence of a coronal plane deformity, presence of significant fragmentation and the scapholunate angle. The presence of coronal/sagittal deformity or significant comminution was a subjective observer-based decision, i.e. the observer made a subjective decision as to whether any coronal or sagittal deformity and whether comminution was present in binary terms, no quantifiable metric was used.

**Fig. 1:**
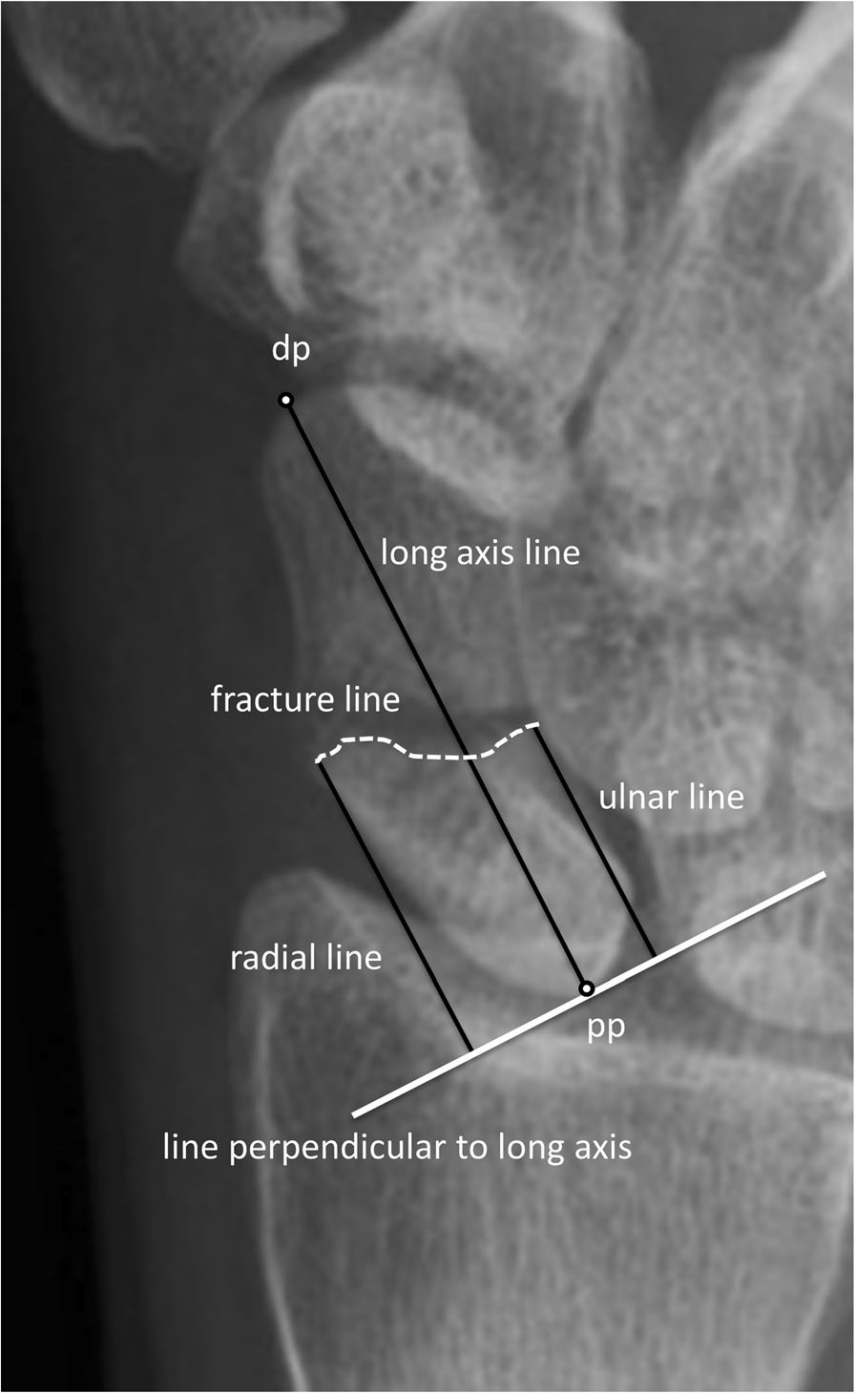
Plain radiograph with the key points relating to ‘long axis’ measurement marked. Detailed legend - a scaphoid fracture with the distal point (dp), proximal point (pp), the long axis line, the fracture line, a line perpendicular to the long axis, and the radial and ulnar lines

**Fig. 2:**
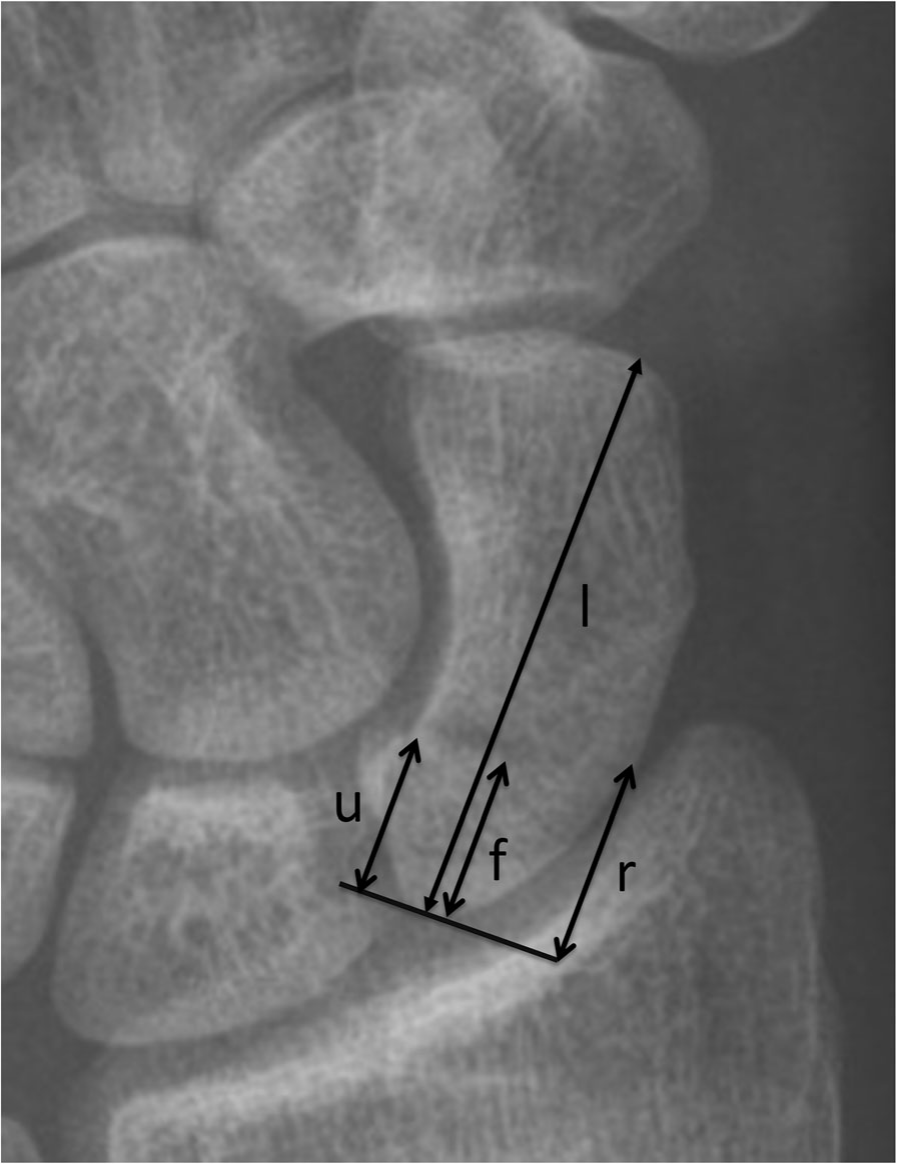
Plain radiograph of a scaphoid fracture demonstrating the measurements made. Detailed legend - the long axis (l) and the distance along the long axis to the fracture site are shown (f, fracture distance), while the ulnar (u) and radial (r) distances to the fracture site are also shown. Note (f) is measured along (l) but for ease of demonstrating the methodology (f) has been moved just adjacent to (l). In this example the long axis measurement is f/l which equals 0.28

The classification and characteristics were recorded separately at different times for both plain radiographs and CT scans. The long axis length of scaphoid was measured from the most proximal ulnar corner of the scaphoid to the centre of the scaphoid tubercle distally (distal point (dp)) (Figs. 1 and 2).

The radiographs were analysed pragmatically with the specific (long axis/radial/ulnar) measurements taken from what was deemed the best long axis view by each observer. The CT scans were analysed using InSight PACS (Insignia medical systems, UK) and the scaphoid orientated to obtain the best long axis view in the opinion of the observer for the specific measurements in this plane. The fracture position was measured using the mid-sagittal coronal image. One observer at each centre repeated the X-ray and CT based assessments six months later to test intra-observer reliability.

### Statistics

Statistical analysis was carried using SPSS version 24 for Windows (IBM Corp). Data was normally distributed unless otherwise stated. Results are expressed as mean (SD) unless otherwise stated. Inter-observer reliability was determined for ordinal data using Cohen’s Kappa and for continuous data using the Intraclass Correlation Coefficient (ICC). ICCs were denoted as the single measures (average measures). Statistical significance was set at a level of *p* < 0.05. The interpretation of the degree of agreement determined by the Kappa and ICC is generally graded as slight (0.01–0.2), fair (0.21–0.40), moderate (0.41–0.60), substantial (0.61–0.80) and almost perfect (> 0.81) [9]. When calculating the ICC for more than two observers the ICC was re-calculated; whereas for the Kappa statistic a mean was used when an overall value was calculated for more than two observers. We did not carry out a formal power calculation, as this is not standard practice for reliability studies [10]; however our sample size and number of observers was comparable with the best practice described within the literature [11].

## Results

### Patient demographics

Table 1 depicts the basic patient demographics including age, sex and the times from injury to imaging. The mean patient age was close to 30 years and a large majority of patients were male in both centres. All imaging was carried out within a month of injury.

**Table 1:** Patient demographics and injury details

	Centre 1	Centre 2
Patient number	30	30
Age	31.4 (11.3)	29 (13.9)
Sex	27 M/3 F	25 M/5 F
Median time from injury to Xrays in days (IQR)	0 (0–7)	0 (0–5)
Median time from injury to CT in days (IQR)	6.5 (5–13)	12.5 (6.5–21)

### Inter-observer reliability of X-ray based results

**Fig. 3:**
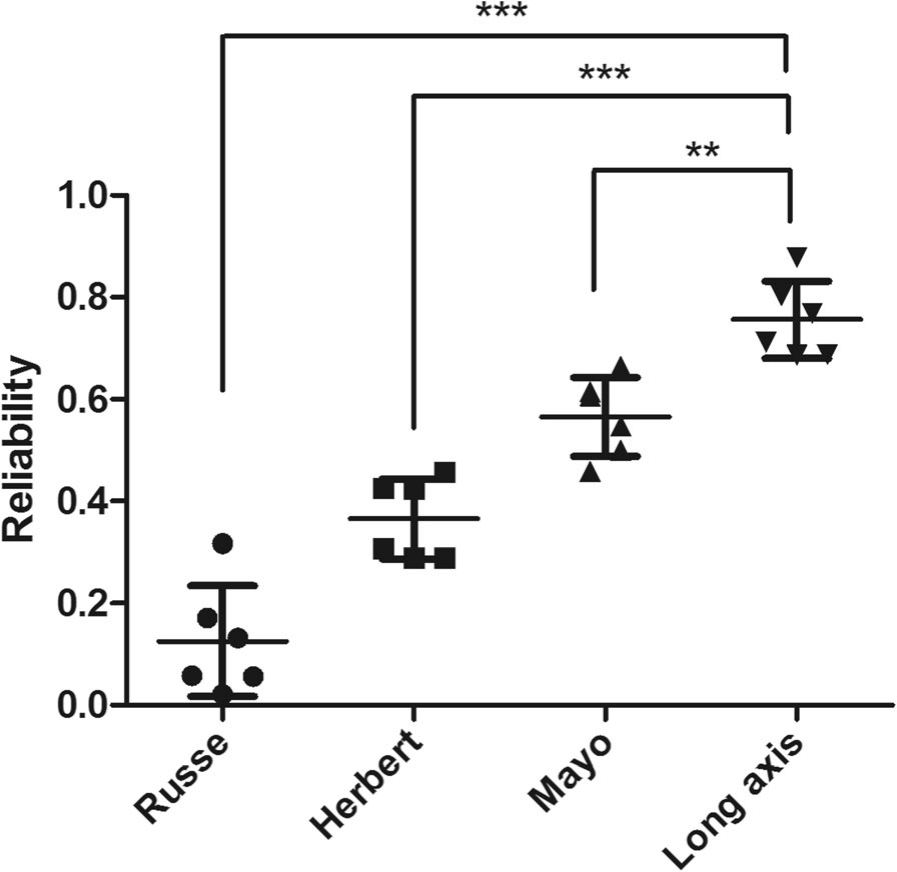
Scatter plot depicting the reliability of the ‘long axis’ measurement in comparison with the more established classification systems. Detailed legend – this demonstrates the reliability of the Russe, Herbert, Mayo and long axis systems. As tested using Bonferroni’s multiple comparison test the long axis measurement system was significantly more reliable than the Mayo (*p* < 0.01), the Herbert (*p* < 0.001) and the Russe (*p* < 0.001) classification systems

Table 2 and Appendix 1 show the inter observer reliability of the all X-ray based assessments. The Mayo classification system was the most reliable of the established classification systems with moderate agreement (kappa = 0.566), with the Herbert and Russe systems demonstrating fair and slight agreement respectively. The long axis measurement demonstrated substantial agreement (ICC = 0.758) and was more reliable than the ulnar and radial measurements. The degree of agreement of measuring sagittal plain deformity was poor (ICC = 0), while the degree of agreement for coronal plain deformity; comminution and scapholunate angle was moderate.  The reliability of the ‘long axis’ measurement versus established classification systems is shown in Fig. 3, with the 'long axis' measurment demonstrating significantly greater reliability than the established methods.

**Table 2:** Inter observer reliability of the X-ray based measurements

System	Centre 1	Centre 2	Overall mean
Russe	0.131	0.120	0.126
Herbert	0.345	0.386	0.366
Mayo	0.609	0.522	0.566
Long axis	0.732	0.784	0.758
Sagittal deformity	−0.02	0.0167	0.00
Coronal deformity	0.425	0.438	0.432
Comminution	0.410	0.529	0.470
Scapholunate angle	0.389	0.580	0.485

### Inter-observer reliability of CT based results

Table 3 and Appendix 2 show the reliability of each tool when using CT. Just as with plain radiographs the Mayo classification system was the most reliable of the established classification systems with moderate agreement (kappa = 0.542), with the Herbert and Russe systems demonstrating fair and slight agreement respectively. Long axis measurement demonstrated substantial agreement (ICC = 0.701); measuring using the radial or ulnar border of the scaphoid was less reliable than the central axis. Reliability was lower for CT measurements than those made based on X-rays. However the reliability of assessment of sagittal deformity (ICC = 0.201) and comminution (ICC = 0.525) was better on CT than on plain radiographs.

**Table 3:** Inter observer reliability of the CT based measurements

System	Centre 1	Centre 2	Overall mean
Russe	0.085	0.055	0.070
Herbert	0.389	0.308	0.348
Mayo	0.531	0.553	0.542
Long axis	0.715	0.686	0.701
Sagittal deformity	0.210	0.192	0.201
Coronal deformity	0.212	0.240	0.226
Comminution	0.328	0.722	0.525

### Relationship between X-ray and CT based results

The degree of agreement between the X-ray and CT based assessments are shown in Table 4 and Appendix 3. The Mayo demonstrated substantial agreement (kappa = 0.791) compared to the moderate agreement of the Herbert (kappa = 0.591) and the fair agreement of the Russe (0.217). The long axis measurement demonstrated moderate agreement (ICC = 0.571).

**Table 4:** The degree of agreement between X-ray and CT based measurements

System	Centre 1	Centre 2	Overall mean
Russe	0.027	0.407	0.217
Herbert	0.506	0.676	0.591
Mayo	0.778	0.799	0.788
Long axis	0.594	0.548	0.571
Sagittal deformity	0.251	0.274	0.262
Coronal deformity	0.339	0.355	0.347
Comminution	0.436	0.325	0.381

### Intra-observer reliability of X-ray and CT based results

The data describing the inter-observer reliability is shown in Table 5 and Appendix 4. As regards X-ray based assessment, the Mayo classification system showed the highest inter-observer reliability of the established classification systems with almost perfect agreement (kappa = 0.824), with the Herbert and Russe systems both demonstrating substantial agreement. The long axis measurement demonstrated almost perfect agreement whether based on X-ray (ICC = 0.895) or CT (ICC = 0.889).

**Table 5:** Intra observer reliability of the X-ray and CT based measurements: the ‘long axis’ measurement versus established classification systems.

System	Xray	CT
Russe	0.583	0.550
Herbert	0.694	0.839
Mayo	0.824	0.855
Long axis	0.895	0.889

## Discussion

A simple ‘long axis’ measurement of the relative distance of the fracture site along the long axis of the scaphoid demonstrates a substantial level of inter observer reliability which is significantly better than other scaphoid classification systems. We found The Mayo to be the most reliable of among popular scaphoid classification systems with a moderate level of inter observer reliability. The new ‘long axis’ metric can be reliably measured on both X-ray and CT, while its other significant advantage over other classification systems is that it provides a way of quantifying fracture position with significant potential benefits for use in clinical research.

Our results are consistent with previous work in this area showing limited reliability of traditional methods [3, 12, 13]. Desai et al. demonstrated that the Russe and Herbert systems had fair levels of agreement, similar to the level of reliability shown in this study [12], while assessments of fracture level, comminution and displacement showed moderate inter- and intra-observer reproducibility [12]. Bhat et al. demonstrated that measures such as the intra-scaphoid angles (sagittal and coronal), the height-to-length ratio and the dorsal scaphoid cortical angle have poor reproducibility [13]. To our knowledge no system for quantifying how relatively proximal or distal an acute fracture has either been created or assessed for reliability, although a method with demonstrable reliability has previously been described relating to scaphoid non-unions [14]. We found that assessing the position of the fracture in relation to the long axis was reliable when measuring from plain radiographs and CT scans. While the use of CT scans is important when measuring union [6] they are not uniformly used in treatment planning initially and it is useful to have a valid measure of fracture position based on plain radiographs alone. This method can be used to allow analysis of a more continuous measure of fracture site and outcome, or alternatively as a tool to allocate fractures into categories such as ‘proximal 20%’ or ‘proximal third’. When publishing raw data for meta-analysis this method might allow research teams to remove and reassign category boundaries. It is also likely that with greater standardisation of methodology the data will become more reliable [10]. It is important to note that this study is one of reliability and not validity. It is not possible to state whether the new ‘long axis’ measurement is ‘better’ than any other method, this study has simply shown that it is more reliable. Certainly, future research is necessary to demonstrate that the ‘long axis’ measurement is of real clinical meaning, for example in predicting the likelihood of scaphoid fracture union.

### Strengths and limitations

This study was pragmatic in terms of how the ‘long axis’ measurement should be performed and yet reliability was high. We would therefore expect reliability of the ‘long axis’ method to be applicable to other centres. There was a range in the level of experience of the observers taking part in the study. We feel that a variable level of seniority and experience gives more generalisable results which easily translate into clinical practice more realistically; any method of assessing acute scaphoid fractures should be simple to use and not require extensive levels of experience. The slightly poorer reliability of the long axis measurement on CT versus X-ray may be related to the freedom of the instructions given to the observers in terms of how to perform this calculation; it may be that future attempts to calculate a long axis measurement on CT need to be more specific and detailed in terms of precisely how observers should go about this.

The most important strength of this study is that the ‘long axis’ method enables the quantification of fracture position. A limitation of the long axis measurement is that it does not describe any information regarding the obliquity of the fracture in any plane, therefore fractures that are particularly oblique to the long axis in the coronal plan may extend deceptively proximally and this will not be communicated by using the central long axis measurement in isolation. It would be possible to calculate the approximate obliquity of the fracture plane using the long axis, radial and ulnar measurements; this is a feasible area for future research. There are also options of using three dimensional CT reconstructions define the fracture plane in multiple dimensions, relative to the long axis.

## Conclusions

This study describes a novel pragmatic ‘long axis’ method for the quantification of acute scaphoid fractures which demonstrates substantial inter and intra observer reliability. The ‘long axis’ method offers benefits over traditional classification in terms of reliability, allocation of fractures to anatomical subgroups, and contributing to future debate on the definition and implications of proximal pole injuries.

### Clinical relevance

- The most widely used acute scaphoid classification systems are not particularly reliable and do not quantify the approximate fracture location
- A simple ‘long axis’ measurement of the relative distance of the fracture site along the long axis of the scaphoid demonstrates significantly better inter observer reliability than the widely used classification systems
- This novel ‘long axis’ metric can be reliably measured on both plain radiographs and CT, while it provides a way of reliably quantifying fracture position which has significant potential uses in future clinical research

## Appendix 1

**Table 6:** Xray based reliability

	Centre 1				Centre 2				Overall mean
	1 vs 2	1 vs 3	2 vs 3	Mean	1 vs 2	1 vs 3	2 vs 3	Mean	
Russe reliability (Kappa)	0.317	0.021	0.056	0.131	0.171	0.058	0.132	0.120	0.126
Herbert reliability (kappa)	0.289	0.289	0.457	0.345	0.424	0.426	0.308	0.386	0.366
Mayo reliability (kappa)	0.665	0.615	0.548	0.609	0.607	0.459	0.500	0.522	0.566
Central distance reliability (ICC)	0.713(0.832)	0.803(0.891)	0.687(0.815)	0.732(0.891)	0.688(0.815)	0.768(0.869)	0.878(0.935)	0.784(0.916)	0.758(0.904)
Ulnar distance reliability(ICC)	0.657(0.793)	0.857(0.923)	0.650(0.788)	0.709(0.880)	0.664(0.798)	0.359(0.528)	0.765(0.867)	0.590 (0.812)	0.650(0.846)
Radial distance reliability(ICC)	0.656(0.792)	0.785(0.880)	0.708(0.829)	0.716(0.883)	0.581(0.735)	0.600(0.750)	0.385(0.556)	0.523(0.767)	0.620(0.825)
Sagittal deformity reliability(kappa)	−0.119	0.118	−0.05	−0.02	−0.061	0.048	0.063	0.0167	0.00
Coronal deformity reliability(kappa)	0.333	0.250	0.692	0.425	0.659	0.328	0.328	0.438	0.432
Comminution deformity reliability(kappa)	0.268	0.514	0.447	0.410	0.561	0.615	0.412	0.529	0.470
SL angle reliabilityICC)	0.331(0.498)	0.422(0.594	0.435(0.607)	0.389(0.656)	0.688(0.815)	0.449(0.620)	0.629(0.773)	0.580(0.806)	0.485(0.731)

## Appendix 2

**Table 7:** CT based reliability

	Centre 1				Centre 2				Overall mean
	1 vs 2	1 vs 3	2 vs 3	Mean	1 vs 2	1 vs 3	2 vs 3	Mean	
Russe reliability(Kappa)	0.00	0.253	0.00	0.084	0.066	0.056	0.156	0.055	0.070
Herbert reliability(kappa)	0.464	0.389	0.314	0.389	0.271	0.425	0.227	0.308	0.348
Mayo reliability(kappa)	0.562	0.568	0.463	0.531	0.717	0.462	0.481	0.553	0.542
Central distance reliability(ICC)	0.748(0.856)	0.687(0.814)	0.701(0.825)	0.715(0.883)	0.636(0.717)	0.569(0.725)	0.600(0.750)	0.686(0.867)	0.701(0.875)
Ulnar distance reliability(ICC)	0.667(0.800)	0.664(0.798)	0.476(0.645)	0.598(0.817)	0.400(0.572)	0.359(0.528)	0.178(0.302)	0.307(0.571)	0.453(0.694)
Radial distance reliability(ICC)	0.650(0.788)	0.717(0.835)	0.569(0.725)	0.644(0.844)	0.455(0.625)	0.596(0.747)	0.224(0.366)	0.437(0.699)	0.541(0.772)
Sagittal deformity reliability(kappa)	0.400	0.067	0.163	0.21	0.366	0.098	0.112	0.192	0.201
Coronal deformity reliability(kappa)	0.268	0.118	0.250	0.212	0.420	0.189	0.112	0.240	0.226
Comminution deformity reliability(kappa)	0.279	0.279	0.426	0.328	0.684	0.815	0.667	0.722	0.525

## Appendix 3

**Table 8:** Xray versus CT reliability

	Centre 1				Centre 2				Overall mean
	1	2	3	Mean	1	2	3	Mean	
Russe reliability(Kappa)	0.00	0.00	0.082	0.027	0.054	0.224	0.944	0.407	0.217
Herbert reliability(kappa)	0.705	0.412	0.402	0.506	0.544	0.586	0.898	0.676	0.591
Mayo reliability(kappa)	0.918	0.627	0.790	0.778	0.904	0.804	0.688	0.799	0.788
Central distance reliability(ICC)	0.724(0.840)	0.464(0.634)	0.593(0.744)	0.594(0.739)	0.767(0.868)	0.427(0.599)	0.451(0.621)	0.548(0.696)	0.571(0.718)
Ulnar distance reliability(ICC)	0.826(0.905)	0.349(0.517)	0.573(0.728)	0.583(0.717)	0.449(0.620)	0.465(0.635)	0.161(0.278)	0.358(0.511)	0.471(0.614)
Radial distance reliability(ICC)	0.719(0.837)	0.467(0.637)	0.784(0.879)	0.657(0.784)	0.751(0.858)	0.393(0.565)	0.271(0.427)	0.472(0.617)	0.565(0.700)
Sagittal deformity reliability(kappa)	0.267	0.217	0.270	0.251	0.405	0.05	0.366	0.274	0.262
Coronal deformity reliability(kappa)	0.634	0.333	0.050	0.339	0.189	0.772	0.105	0.355	0.347
Comminution deformity reliability(kappa)	0.298	0.585	0.426	0.436	0.815	0.615	0.359	0.325	0.381

## Appendix 4

**Table 9:** Inter observer reliability for X-ray and CT based assessments

	Observer 1		Observer 2		Mean	
	X-ray	CT	X-ray	CT	X-ray	CT
Russe reliability(Kappa)	0.628	0.679	0.538	0.420	0.583	0.550
Herbert reliability(kappa)	0.749	0.850	0.639	0.828	0.694	0.839
Mayo reliability(kappa)	0.831	0.838	0.817	0.871	0.824	0.855
Central distance reliability(ICC)	0.905(0.950)	0.900(0.948)	0.884(0.938)	0.878(0.935)	0.895(0.944)	0.889(0.946)
Ulnar distance reliability(ICC)	0.820(0.901)	0.920(0.958)	0.568(0.725)	0.839(0.912)	0.694(0.813)	0.807(0.890)
Radial distance reliability(ICC)	0.822(0.902)	0.931(0.964)	0.850(0.919)	0.755(0.860)	0.836(0.911)	0.843(0.912)
Sagittal deformity reliability(kappa)	0.765	0.891	0.612	0.636	0.689	0.764
Coronal deformity reliability(kappa)	0.818	0.826	0.433	0.622	0.626	0.724
Comminution deformity reliability(kappa)	0.774	0.840	0.689	0.944	0.732	0.892

## Abbreviations

CT: Computed tomography
ICC: Intraclass Correlation Coefficient
